# Modification of polyethersulfone membrane using MWCNT-NH_2_ nanoparticles and its application in the separation of azeotropic solutions by means of pervaporation

**DOI:** 10.1371/journal.pone.0236529

**Published:** 2020-07-22

**Authors:** Azam Marjani, Ali Taghvaie Nakhjiri, Maryam Adimi, Hassan Fathinejad Jirandehi, Saeed Shirazian

**Affiliations:** 1 Department for Management of Science and Technology Development, Ton Duc Thang University, Ho Chi Minh City, Vietnam; 2 Faculty of Applied Sciences, Ton Duc Thang University, Ho Chi Minh City, Vietnam; 3 Department of Petroleum and Chemical Engineering, Science and Research Branch, Islamic Azad University, Tehran, Iran; 4 Department of Chemical Engineering, Farahan Branch, Islamic Azad University, Farmahin, Farahan, Iran; 5 Department of Chemistry, Farahan Branch, Islamic Azad University, Farmahin, Farahan, Iran; 6 Institute of Research and Development, Duy Tan University, Da Nang, Vietnam; 7 The Faculty of Environmental and Chemical Engineering, Duy Tan University, Da Nang, Vietnam; Brandeis University, UNITED STATES

## Abstract

In this study, functionalized multi-walled carbon nanotubes (MWCNT-NH_2_) were synthesized as an additive for the preparation of mixed matrix membranes (MMMs) and then were investigated by FTIR and FE-SEM techniques. Polyether sulfone (PES) polymeric membrane modified with functionalized MWCNT-NH_2_ carbon nanotubes was prepared by phase inversion method. The effect of MWCNT-NH_2_ on the morphology and property of the PES membrane was evaluated using scanning electron microscopy. The flux, enrichment factor and swelling properties of modified membranes were also used to investigate the membranes performance. The results showed that the flux and enrichment factor in modified PES membrane containing 5 wt.% of functionalized MWCNT-NH_2_ carbon nanotubes were obtained 1.2 L.m^-2^h^-1^ and 3.3, respectively. The influence of methanol concentration on the flux and enrichment factor was investigated. The results corroborated that the flux didn’t change significantly, while the enrichment factor was decreased.

## 1. Introduction

Membrane processes have an indisputable position in the separation and extraction industries such as gas sweetening, wastewater treatment and food / medical industries [1–6]. Great and irrefutable capabilities such as low investment cost, simple technology, high flexibility in system design and appropriate environmentally friendly characteristics have motivated numerous researchers for further investigations [7–12]. Various membrane separation processes have been designed and developed so far for purification/separation of gas and liquid mixtures [13–19].

Pervaporation is a dual separation process. It means that both mass transfer and heat transfer occur and are essentially used to separate two liquid components, in which the percentage of one liquid is much lower than the other. One of the main applications of pervaporation process is separation of the azeotropic mixtures [20]. The azeotrope prevents optimum purification of the organic materials. Azeotropic distillation is usually used to overcome this problem. However, this process possesses high operational cost due to the need to apply a third solvent and then to remove it at the end of process. But evaporation can conveniently solve this problem. Since the pervaporation process can be carried out at ambient temperatures or temperatures below normal distillation, it is a beneficial alternative to conventional distillation [21–23]. Another advantage of pervaporation process is its low cost due to the operation at low temperature. This process requires less operating space compared to distillation or other separation methods because the separation is implemented by a membrane with a thickness of several microns [24, 25].

Carbon nanotubes are regarded as one of the most extensively used fillers applied in the nanocomposites preparation [26]. Most single-walled carbon nanotubes have a diameter of about 1 nm, but their length may be several thousand times bigger than their diameter. In fact, the single-walled carbon nanotubes are the wrapped and tubular state of graphene. These structures have much stronger electrical properties than multiwall state [27–29]. Laminated carbon nanotubes consist of several layers of graphite that are wrapped in a tubular state. For this reason (larger pieces of multi-walled carbon nanotubes), these structures are more fragile than single-wall modes [30]. Single-walled and multi-walled carbon nanotubes have been widely used as reinforcing agents in the preparation of a variety of polymer nanocomposites, largely due to their superior thermal and mechanical properties [27, 28]. Therefore, incorporation of carbon nanotubes in polymeric membranes can provide better separation properties for purification of liquid mixtures.

Asymmetric polyamide membranes were prepared by Abdallah et al. via reverse phase method and used to separate methanol from methyl acetate. The results showed that increment of the temperature declined the separation factor and increased the flux. Additionally, they implied that increasing the concentration of methanol in the feed improved the separation factor and the flux [31]. Liu et al. separated thiophene from normal heptane applying polyether block amide (PEBA) and polyvinylidene fluoride (PVDF) composite membranes. They perceived that increase in the polyamide and polyether amounts hardened the PEBA structure and enhanced the amorphous state in the polymer and the free volume, respectively [32]. Pakizah et al. prepared a new PSF / Pebax / F-MWCNTs nanocomposite membrane and used it for oil / water emulsion nanofiltration. For this purpose, a porous PSF backbone was prepared and then a thin layer of Pebax was coated on it as the selective layer. The effect of adding 0.5, 1 and 2 wt. % of multiwall carbon nanotubes (F-MWCNTs) on the morphological and separation properties of the prepared membranes was systematically studied. The results proved that increase in the F-MWCNT to 2 wt. % increased the hydrophilicity, tensile strength, and thermal stability [33].

The prominent objective of this research paper is to synthesize the functionalized MWCNT-NH_2_ carbon nanotubes for the mixed matrix membrane preparation. Moreover, FTIR and FE-SEM analyses are implemented to investigate the structure of prepared functionalized MWCNT-NH_2_ carbon nanotubes. Phase separation method is then applied to fabricate modified polyethersulfone (PES) polymeric membrane with functionalized MWCNT-NH_2_ carbon nanotubes. Additionally, the effect of MWCNT-NH_2_ filler on the morphology and characterization of PES membrane is investigated by scanning electron microscopy (FE-SEM).

## 2. Experimental and methodology

### 2.1. Synthesis of functionalized MWCNT-NH_2_ carbon nanotubes

For this purpose, 5 gr of raw MWCNT (supplied from Nanoshel Co., USA) was added to 100 ml H_2_SO_4_ / HNO_3_ mixture (purchased from Merck Co.) at a volume ratio of 1:3 and thoroughly dispersed by ultrasonic for 20 min at room temperature. Then, the homogenous mixture was connected to the reflux system at 85°C for 24 h. The resulting product was then filtered and washed with deionized water until achieving pH of 7, and then dried. In the second step, the obtained MWCNT-COOH was dispersed in 5 ml of thionyl chloride (TCl) under reflux at 60°C for 12 h. Unreacted residual thionyl chloride was removed completely by drying the final precipitate. In the third step, the prepared MWCNT-COCl was mixed with 5 ml of ethylenediamine (EDA) to replace the amine groups on the MWCNT surface. Finally, the obtained product was washed and dried with ethanol.

### 2.2. Preparation of the PES / functionalized MWCNT-NH_2_ nanocomposite membranes

As presented in Table 1, to prepare the desired membranes, appropriate amounts of functionalized carbon nanotubes were mixed with PES polymer (supplied by Sigma-Aldrich Co., USA) and solvent, and stirred in a closed container at 80°C until complete dissolution of the polymer. After dissolving the polymer, the desired solution was heated without stirring with the aim of removing the bubbles at the same temperature. Then, the membrane was slowly immersed in distilled water to be precipitated. The membrane remained in the water for 24 h to be applied for the separation processes.

**Table 1:** The amount of various components for membranes preparation.

Sample	Filler to polymer weight (%)	MWCNT-NH_2_ carbon nanotubes (gr)	PES polymer (gr)	NMP solvent (gr)
**M0**	0	0	2	8
**M1**	3	0.3	2	7.7
**M2**	5	0.5	2	7.5
**M3**	7	0.7	2	7.3

### 2.3. Experimental setup

**Fig 1:**
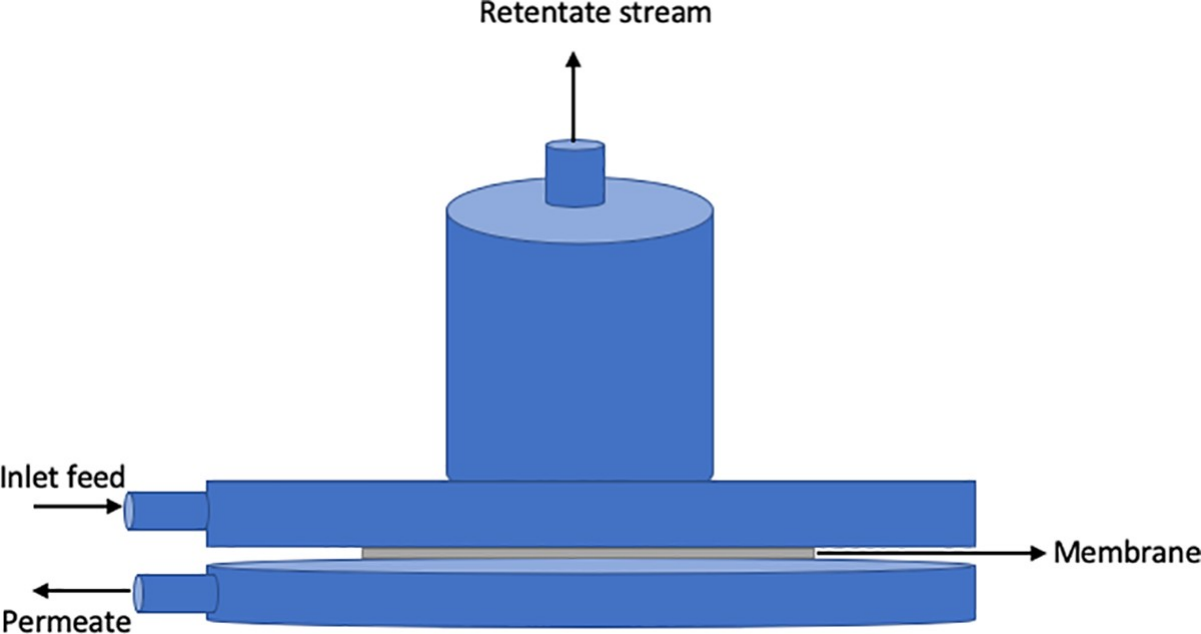
Schematics of membrane module used in this study.

Fig 1 illustrates the schematic representation of lab-scale pervaporation module applied for conducting the separation tests. The setup contains the following sections:

1. A membrane module made up of two upper and lower parts, which are connected by four screws. An appropriate O-ring is placed at the lower part of the module to seal the groove. At the outlet of the cell, a reticular plate is provided for mechanical protection of the membrane. Membrane cell is made of stainless steel due to its high chemical resistance. The feed comes into contact with the membrane after crossing a channel parallel to the membrane surface. The membrane with a diameter of 3.5 cm is located in the lower half of the module on the metal support.
2. Heaters (L81ST model, Labinco Co., Netherlands) and hot water baths (Memmert Co., Germany) to regulate feed temperature in set point.
3. Digital temperature control system (DHS22 model, Pals electronic system Co., Iran) to control the feed temperature.
4. A vacuum pump (PM200 model, Memmert Co., Germany) to create a vacuum equal to 0.2 bar.
5. A liquid nitrogen reservoir to condense the permeated vapors.

After inserting the membrane into the module and assembling the fittings, the feed is circulated by means of a pump with a flow rate of 2 L.min^-1^ from the tank containing 1 L of feed. All experiments have been conducted in duplicate, and the average values are reported.

The flux is calculated as follows [34]:

$$J=\frac{Q}{A\times t}$$ (1)

In this equation *J*, *Q*, *A* and *t* are interpreted as the flux, the amount of fluid permeated (L), cross-sectional area (m^2^) and time (h), respectively. The enrichment factor (*β_i_*) can be interpreted as a ratio between the compound’s concentration in the permeate (*c_p,i_*) to the compound’s concentration in the feed (*c_f,i_*) and can be denoted by the following equation [35]:

$$\beta_{i}=\frac{c_{p,i}}{c_{f,i}}$$ (2)

### 2.4. Swelling test

Membranes were cut in 1×1 cm^2^ and placed at room temperature in a solution containing water and methanol, respectively. The samples were weighed at a time interval of 1 h after removal from the solution and the surface cleanliness. After weighing the samples for five times and ensuring no change in the weight of the samples, the samples were left in solution for 72 h. The amount of swelling can be calculated by the following equation:

$$Swelling\left(\%\right)=\frac{w_{s}-w_{d}}{w_{d}}\times 10$$ (3)

where *w*_*s*_ and *w*_*d*_ denote weight of wet and dry sample, respectively.

### 2.5. Preparation of membrane samples for FESEM analysis and contact surface angle measurements

Membrane samples were fractured with liquid nitrogen to appropriate size and sent to apparatus section for FESEM analysis (HITACHI S-4160, operating 30kV). The conductivity of the sample surface is regarded as an important prerequisite for the sample to be examined. Therefore, for non-conductive samples, a thin conductive coating is applied on the sample. In order to analyze the contact surface angle, several water drops were first dropped on each of the prepared membrane applying syringe. The membrane sides were then photographed using the camera and the contact angle of each membrane was determined using *Image J* software.

## 3. Results and discussion

### 3.1. FTIR spectra of functionalized MWCNT-NH_2_ carbon nanotubes

**Fig 2:**
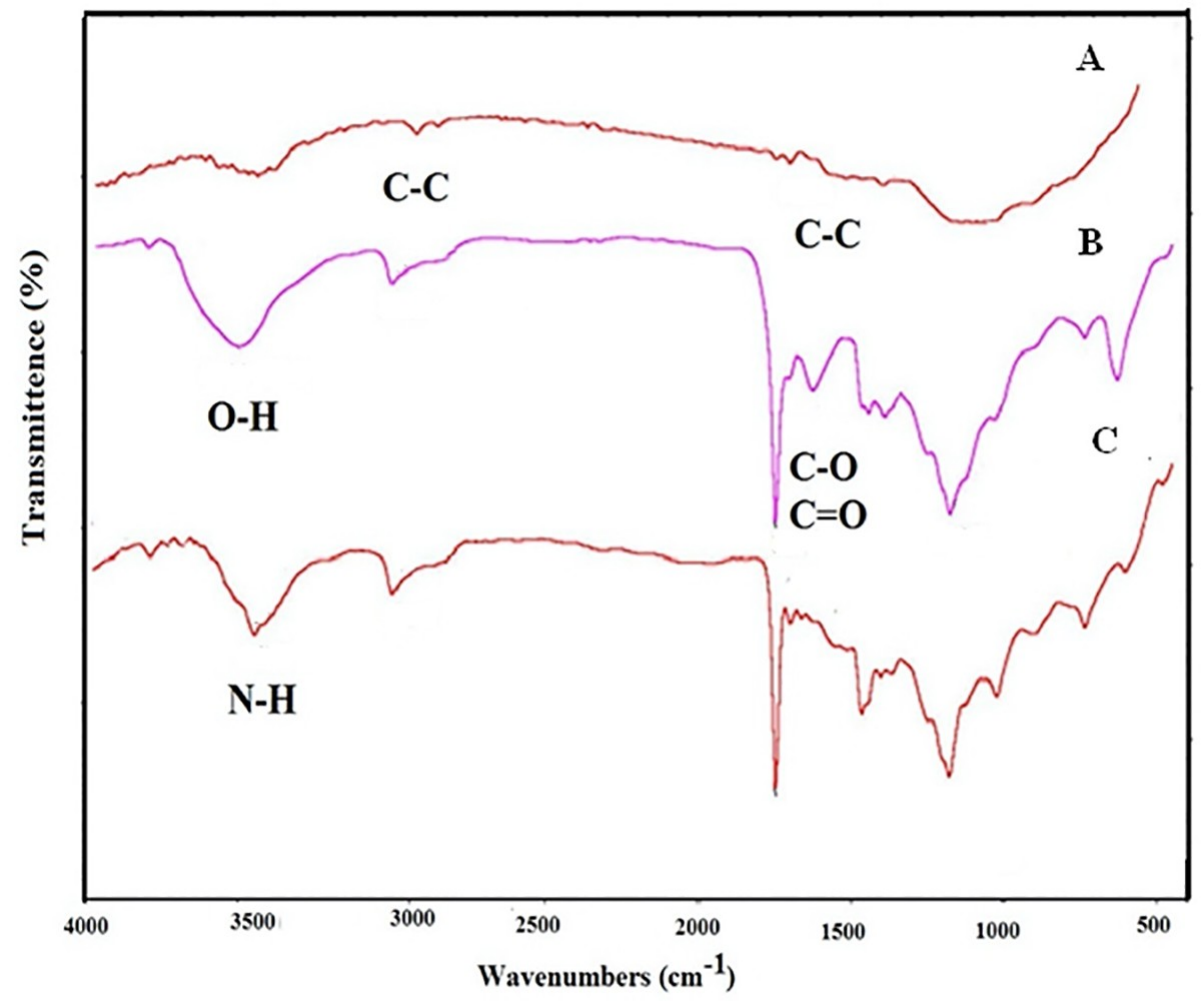
FTIR spectra for carbon nanotubes and functionalized MWCNT-NH_2_ carbon nanotubes.

The FTIR analysis was performed to investigate the functionalization process of MWCNT. Fig 2 illustrates the FTIR analysis of the different stages of MWCNT-NH_2_ synthesis. Pure MWCNT, MWCNT-NH_2_ and MWCNT-COOH spectra are represented in Fig 2. Spectrum A corresponds to the prepared sample from pure MWCNT in which two absorption peaks at 1680 and 3010 cm^-1^ exist that belong to the carbon-carbon tensile vibrations. Spectrum B shows the FTIR spectrum of MWCNT-COOH. The tensile vibrations of the -OH group are seen as a broad peak at 3200–3600 cm^-1^ and the vibrational peak at 1050 cm^-1^ is related to the destruction of the -OH group. In addition, peaks at 1750 cm^-1^ belong to the C-O and C = O bonds. Spectrum C belongs to MWCNT-NH_2_ as the final product. The specific peak seen in the 3400–3500 cm^-1^ region is related to type II amide tensile vibrations, which probably overlaps with type II amine tensile vibrations and confirms the presence of NH_2_ groups in the final product.

### 3.2. Analysis of FESEM images of pure carbon nanotubes and functionalized MWCNT-NH_2_ carbon nanotubes

**Fig 3:**
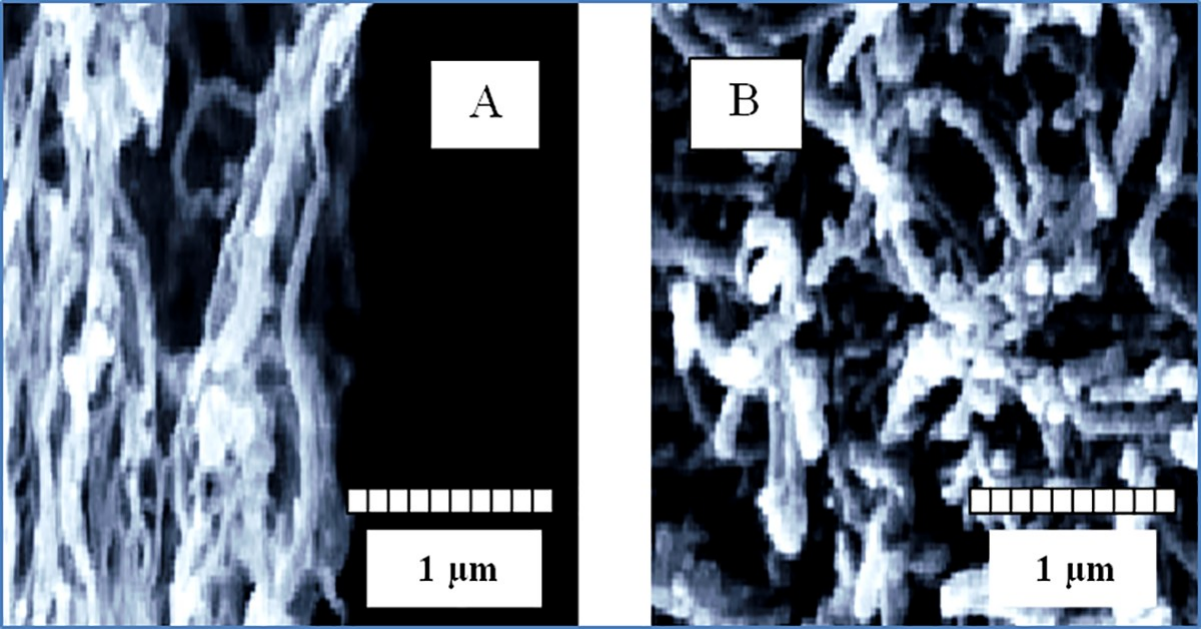
FESEM images of the pure carbon nanotubes (A) and functionalized MWCNT-NH_2_ carbon nanotubes (B).

Fig 3 shows the FE-SEM images of the pure carbon nanotubes and functionalized MWCNT-NH_2_ carbon nanotubes. Due to the application of concentrated acids and high temperatures during the oxidation reaction, the tubular structure is likely to be destroyed. To ensure that the carbon nanotubes remain intact, the SEM image of the materials after oxidation was compared with the SEM image of pure carbon nanotube.

### 3.3. SEM images of PES / MWCNT-NH_2_ composite membranes

**Fig 4:**
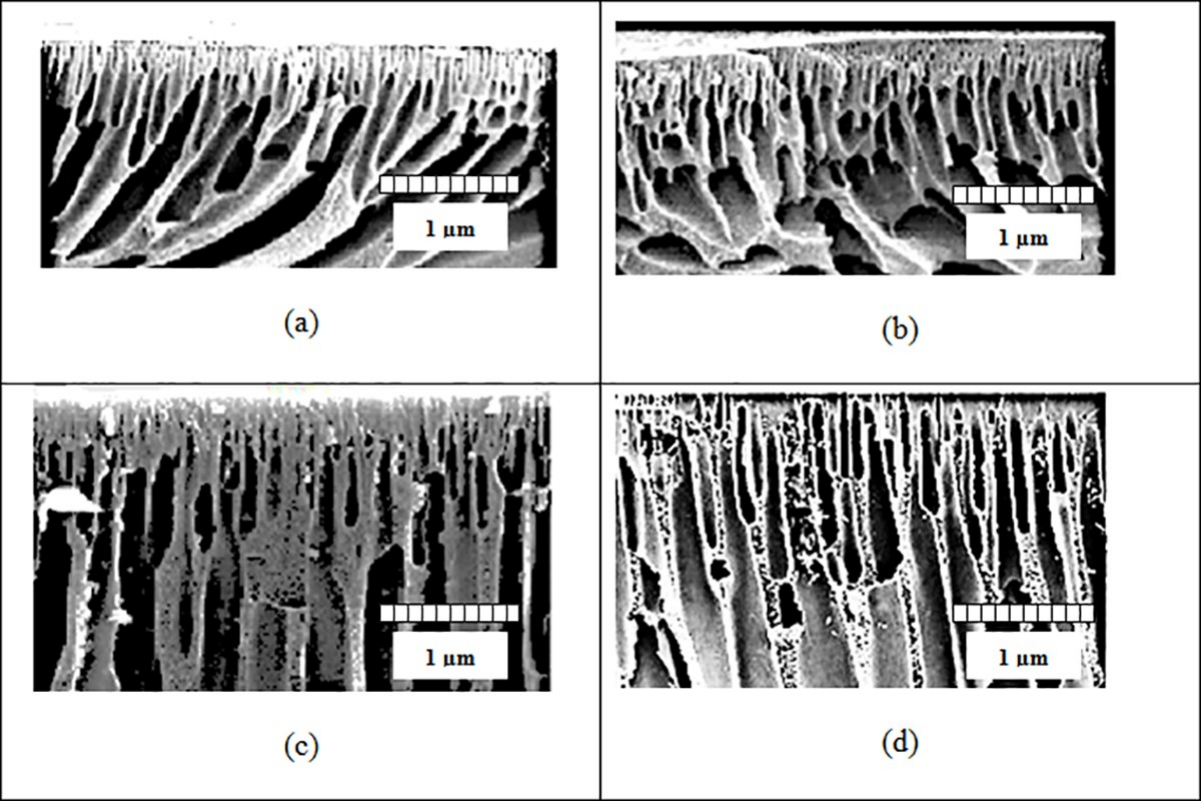
SEM images of PES / MWCNT-NH_2_ composite membranes at different filler percentages.

Fig 4A, 4B, 4C and 4D depict the obtained SEM images from the cross section of prepared membranes containing 0, 3, 5 and 7 wt.% of MWCNT-NH_2_, respectively. All membranes have an asymmetric structure including a compacted upper layer, a middle porous substrate and a bottom layer that are completely deformed and consists of macropores. Neat PES membranes have an asymmetric structure in which the upper layer is compacted and the lower layer has a porous finger-like structure. A marked change in the sublayer of the membranes is significant after the addition of functionalized carbon nanotubes (PES / MWCNT-HN_2_). It is observed tha with the addition of functionalized MWCNT-HN_2_ carbon nanotubes, thickness of the thin layer is decreased.

### 3.4. Analysis of the water contact angle

The membrane hydrophilicity can be determined using the contact angle measurement and is studied to evaluate the membrane function in water/alcohol separation. Smaller contact angle shows higher hydrophilic surface due to the affinity of the materials to water. Table 2 renders the experiments related to water contact angle made on the composite membranes with different percentages of functionalized MWCNT-NH_2_ carbon nanotubes. As seen, it can be claimed that the addition of functionalized MWCNT-NH_2_ carbon nanotubes enhanced the hydrophilic properties of the membranes and consequently decreased the water contact angle, which corroborated the membranes surface alteration by the nanoparticles. Increasing the hydrophilic groups such as MWCNT-NH_2_ to the matrix makes the polymeric membranes more hydrophilic.

**Table 2:** Average contact angle of the prepared membranes.

Sample	Filler percentage (%)	Average contact angle (°)
**M0**	0	86.4
**M1**	3	75.8
**M2**	5	61.4
**M3**	7	41.9

### 3.5. Evaluation of pure water and methanol fluxes for different fabricated membranes

**Fig 5:**
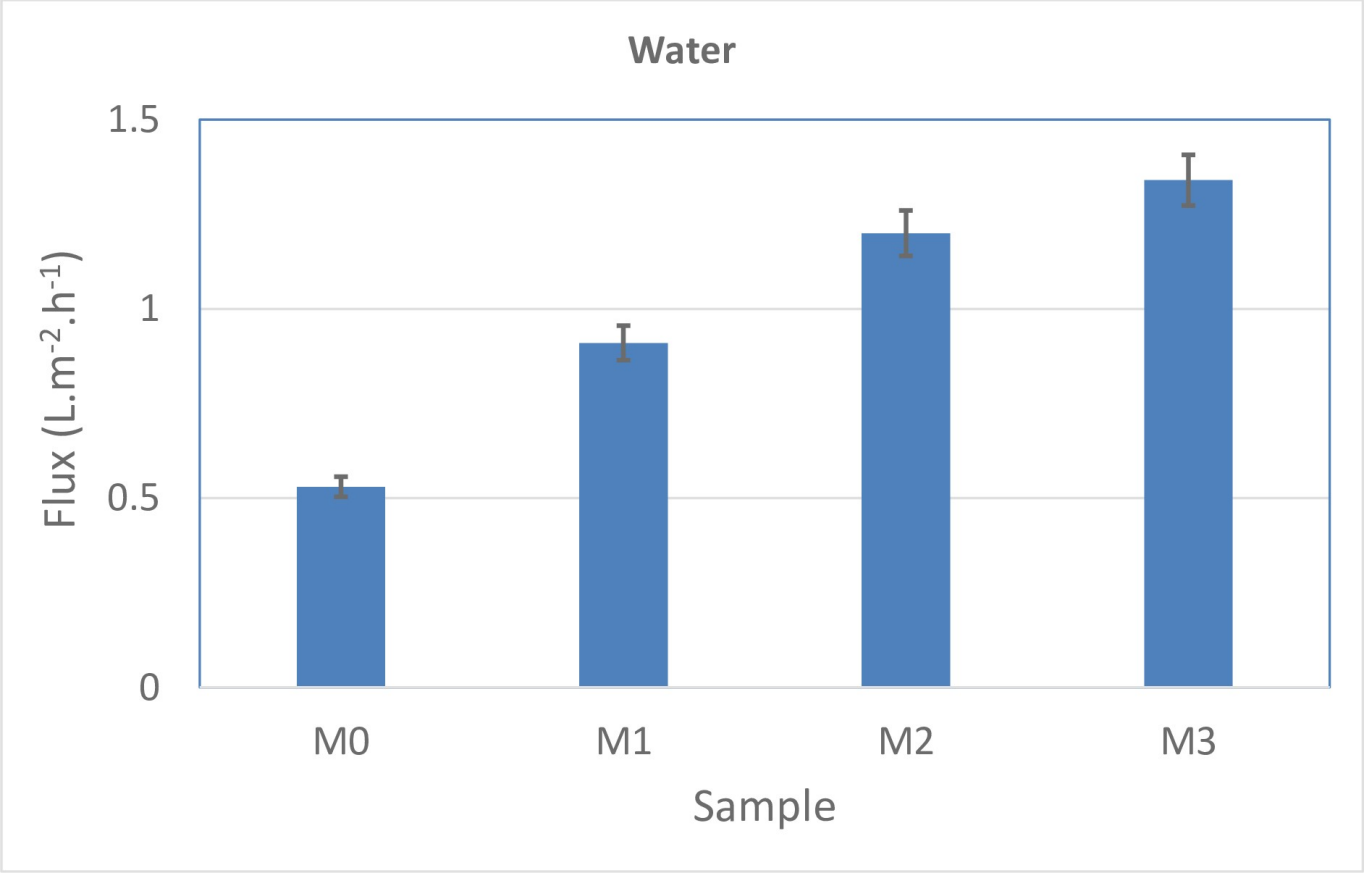
The amount of pure water flux for the fabricated membranes.

The water flux results of the various membranes are shown in Fig 5. The neat membrane exhibits the lowest water flux compared to other membranes. The modified membranes showed an obvious increment in water flux up to 1.34 L.m^-2^.h^-1^. This phenomenon can be justified by increasing of membrane hydrophilicity due to enhancing the amount of MWCNT-NH_2_ hydrophilic component. The highest water flux obtained for M3 membrane (1.34 L.m^-2^.h^-1^). Overally, the pure water flux is improved substantially with increasing the amount of MWCNT-NH_2_ hydrophilic groups in the membrane structure. Moreover, by addition of the MWCNT-NH_2_ filler to PES membrane, the size of pores is increased which eventuated in increasing water flux of the membranes containing fillers.

**Fig 6:**
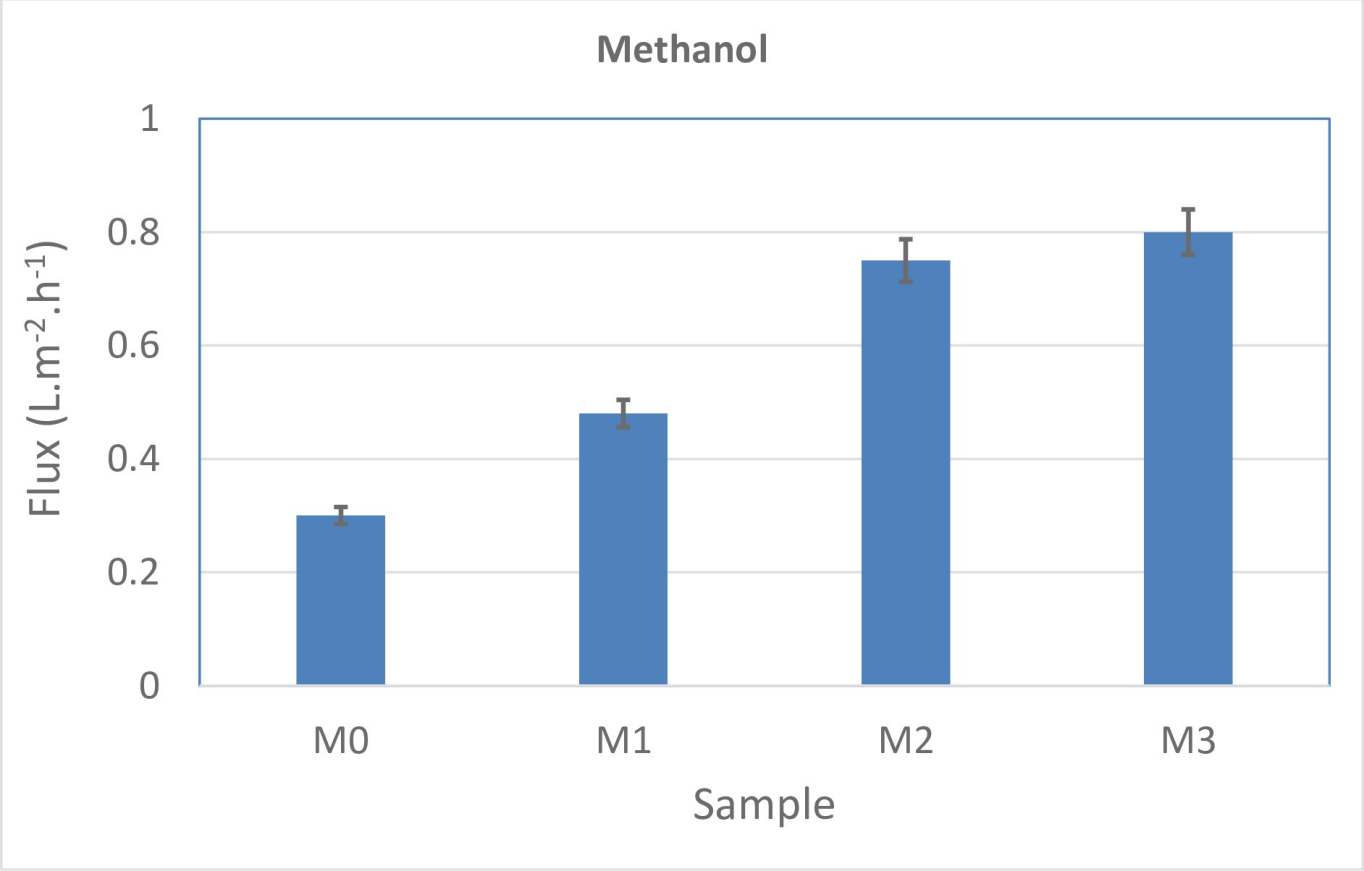
The amount of methanol flux for various fabricated membranes.

The methanol flux results of the prepared membranes are demonstrated in Fig 6. The pure membrane showed the lowest flux of methanol compared to the other membranes, while the modified membranes indicated a significant increase in flux. This behaviour can be justified by increasing membrane porosity due to enhancing the MWCNT-NH_2_ hydrophilic composition. Additionally, compared to the water flux, the methanol flux is lower.

### 3.6. Effect of feed concentration on the performance of modified membranes

The results of concentration tests are presented in Table 3. According to Table 3, at 40 to 60% methanol / water concentration, the enrichment factor droped sharply, so that this value is significantly different in all membranes modified with MWCNT-NH_2_ and pure membrane compared to other methanol / water concentrations. Because of the membrane behavior at this concentration, methanol is more difficult to pass through the membrane than water and its flux is much lower. In other words, water molecules passed through the membrane during testing, but methanol remained behind the membrane, which eventuated in a significant reduction in the amount of methanol passing through the membrane and a lower enrichment factor.

**Table 3:** Influence of feed inlet concentrations on the enrichment factor.

	Enrichment Factor			
Methanol in the feed	M0	M1	M2	M3
85%	1.1	1.5	3.3	1.8
60%	0.9	1.1	2.4	1.3
40%	0.8	0.8	1.3	0.9

### 3.7. Swelling test

**Fig 7:**
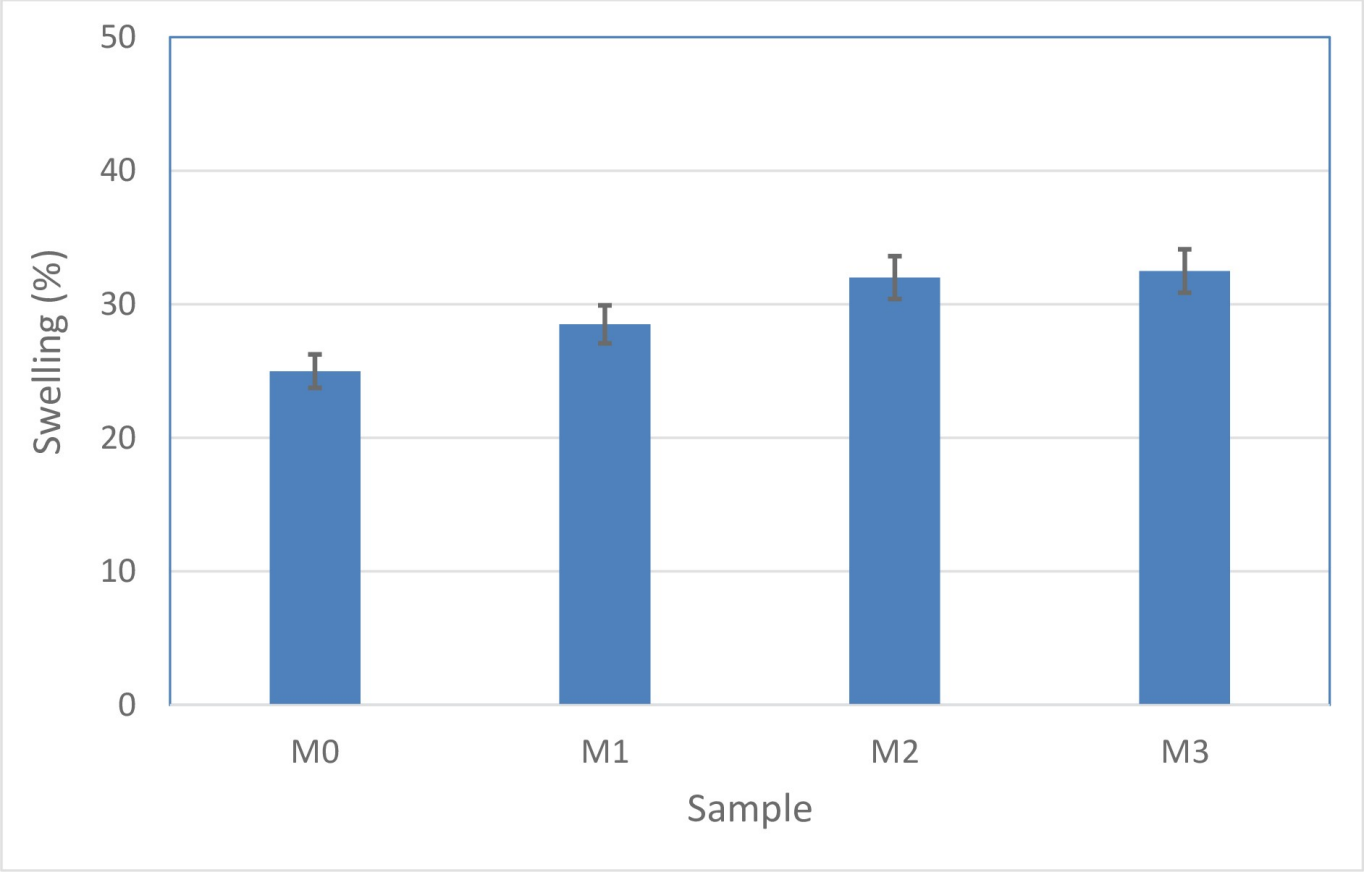
Influence of carbon nanotubes content on swelling of modified membranes.

The results of membrane swelling experiments are depicted in Fig 7. In this analysis, four membranes modified with different percentage of MWCNT-NH_2_ were tested. To reach steady state condition, the membranes were left in the solvent for 72 h and then weighed. The swelling range of all membranes as a function of polar solvent at the ambient temperature is investigated and the results are shown in Fig 7. As shown, the amount of swelling in polar solvent is increased by increasing the MWCNT-NH_2_ weight percentage in the membrane structure which could be attributed to the increasing hydrophilicity and porosity of the membranes.

## 4. Conclusion

In the present investigation, functionalized MWCNT-NH_2_ carbon nanotubes were applied to modify polyether sulfone (PES) membranes. Various analyses were performed to successfully confirm the synthesized cases. The results corroborated that by modifying the membranes, their properties such as water flux, enrichment factor and hydrophilicity have been improved considerably. The FESEM images of modified membranes with functionalized MWCNT-NH_2_ carbon nanotubes showed significant changes in the shape of the channels, pores size and membranes sublayers. Moreover, the results proved that the membrane containing 5 wt.% of functionalized MWCNT-NH_2_ carbon nanotubes as an additive showed superior performance than other membranes with the water flux and enrichment factor of 1.2 L.m^-2^h^-1^ and 3.3, respectively. The effect of methanol concentration on the flux and enrichment factor was investigated. The results showed that although the flux did not change greatly, the enrichment factor was decreased.
